# RECK is not an independent prognostic marker for breast cancer

**DOI:** 10.1186/s12885-015-1666-2

**Published:** 2015-10-08

**Authors:** Luciana R. Gomes, André Fujita, Joni D. Mott, Fernando A. Soares, Leticia Labriola, Mari C. Sogayar

**Affiliations:** 1 Departamento de Bioquímica, Instituto de Química, and NUCEL/NETCEM (Núcleo de Terapia Celular e Molecular), Faculdade de Medicina, Departamento de Clínica Médica, Universidade de São Paulo, Rua Pangaré, 100, São Paulo, 05360-130 SP Brazil; 2 Departamento de Ciência da Computação, Instituto de Matemática e Estatística, Universidade de São Paulo, São Paulo, SP Brazil; 3 Lawrence Berkeley National Laboratory, Life Science Division, Berkeley, CA USA; 4 Departamento de Anatomia Patológica, Hospital A. C. Camargo, Fundação Antônio Prudente, São Paulo, SP Brazil

**Keywords:** RECK, Breast cancer, Immunohistochemistry, Tissue microarray, Biomarker

## Abstract

**Background:**

The REversion-inducing Cysteine-rich protein with Kazal motif (RECK) is a well-known inhibitor of matrix metalloproteinases (MMPs) and cellular invasion. Although high expression levels of RECK have already been correlated with a better clinical outcome for several tumor types, its main function, as well as its potential prognostic value for breast cancer patients, remain unclear.

**Methods:**

The RECK expression profile was investigated in a panel of human breast cell lines with distinct aggressiveness potential. RECK functional analysis was undertaken using RNA interference methodology. RECK protein levels were also analyzed in 1040 cases of breast cancer using immunohistochemistry and tissue microarrays (TMAs). The association between RECK expression and different clinico-pathological parameters, as well as the overall (OS) and disease-free (DFS) survival rates, were evaluated.

**Results:**

Higher RECK protein expression levels were detected in more aggressive breast cancer cell lines (T4-2, MDA-MB-231 and Hs578T) than in non-invasive (MCF-7 and T47D) and non-tumorigenic (S1) cell lines. Indeed, silencing RECK in MDA-MB-231 cells resulted in elevated levels of pro-MMP-9 and increased invasion compared with scrambled (control) cells, without any effect on cell proliferation. Surprisingly, by RECK immunoreactivity analysis on TMAs, we found no association between RECK positivity and survival (OS and DFS) in breast cancer patients. Even considering the different tumor subtypes (luminal A, luminal B, Her2 type and basal-like) or lymph node status, RECK remained ineffective for predicting the disease outcome. Moreover, by multivariate Cox regression analysis, we found that RECK has no prognostic impact for OS and DFS, relative to standard clinical variables.

**Conclusions:**

Although it continues to serve as an invasion and MMP inhibitor in breast cancer, RECK expression analysis is not useful for prognosis of these patients.

## Background

Breast cancer is the first in incidence and the second in mortality among women [1]. Currently, a reduction in overall cancer death rates is due to a combination of earlier diagnosis and treatment improvement [1]. Nevertheless, breast cancer remains a worldwide health problem. The lack of more informative biomarkers to discriminate among patients according to the prognosis and clinical course complicates the choice of therapeutic strategy.

Metastasis development is the main death factor attributed to breast cancer [2]. The organized breakdown of the extracellular matrix (ECM) by matrix metalloproteinases (MMPs) is an essential event in the complex metastatic cascade [3]. Inhibitors of MMPs, known as tissue inhibitors of MMPs (TIMPs), and the membrane-associated MMP inhibitor RECK are also critical for controlling the maintenance of the ECM integrity due to their regulation of MMP function [4–7].

Elevated MMP expression and activity levels have been associated with poor prognosis in several cancer types [8–10]. Surprisingly, a higher expression of MMP inhibitors, such as TIMPs, correlates with a worse outcome for breast cancer patients [11–14]. This ambiguous nature of TIMPs may be due to the wide variety of TIMP functions, which are independent of their activities as MMP inhibitors [6]. However, this finding remains unclear.

RECK, another type of MMP inhibitor, has been shown to decrease cellular invasion [15], most likely by inhibiting the enzymatic activity of MMP-2, MMP-9 and MMP-14 [16, 17]. Additionally, RECK has been shown to decrease MMP-9 mRNA levels [18]. The potential prognostic power of RECK has already been reported for other tumors, such as pancreatic, liver, lung and colorectal cancers [19–23], where RECK expression is inversely correlated with MMP expression during tumor progression [23]. Thus, high expression levels of RECK correlate with a better clinical outcome [23].

However, in breast cancer, the RECK expression profile is controversial and remains unclear [24–27]. Moreover, no functional analysis in human mammary tumors has been previously reported. A retrospective study using 119 cases of breast cancer samples, suggests, similar to other cancer types, that a low expression of RECK indicates a shorter survival rate for patients with invasive breast cancer [24]. The available data indicated that lower RECK mRNA levels were expressed in tumor tissue samples than in normal breast tissue samples, and higher levels were found in invasive human breast cancer cells than in less aggressive ones [24–26].

In the present study, we aimed to evaluate the prognostic significance of RECK in breast cancer patient outcome. The RECK protein expression levels were analyzed in a large series (1040) of breast cancer cases using immunohistochemistry of tissue microarrays (TMAs). Furthermore, we investigated RECK function in mammary neoplasia using RECK-silenced human breast cancer cells, obtained by RNA interference methodology. Here, we present evidence supporting that, although RECK acts as a negative regulator of MMP-9 and invasion, its levels of expression fail to correlate with patient survival rates.

## Methods

### Patients and methods

All breast samples were obtained from patients diagnosed and treated at the A. C. Camargo Hospital, São Paulo, Brazil. TMAs were arranged from a series of 1040 cases of primary breast carcinoma from patients diagnosed between 1980 and 1999. Duplicate 1 mm cores from tumors were used in the TMAs. The median age of the patients was 55 years (range: 26–90 years). The tumor tissues were removed at surgery. The A. C. Camargo Hospital Ethics Committee approved this research (Process number: 1648/12) and waived the need for informed consent term.

Vimentin and pan-cytokeratin were used as tissue immunoreactivity and staining controls. RECK staining was performed using a monoclonal antibody diluted 1:200 (Cell Signaling, Beverly, MA, USA). Several patient and tumor characteristic data were available, including age, menopausal status, tumor size, lymph node stage, SBR stage (Scarff-Bloom-Richardson, histological classification method) and TNM tumor stage. The expression state of several biomarkers, such as estrogen receptor (ER), progesterone (PR), Her-2 and cytokeratins (CK5, CK6, CK8, CK14 and CK18), was also evaluated by two distinct pathologists.

The ScanScope XT device was used for slide scanning. The percentage of cells positively stained for RECK, and its staining pattern in the TMA core, was determined using the Spectrum Plus computational program, by a trained person. For each subject, at least three measurements were obtained, from two distinct slides, with samples represented in duplicate. The average of these three values was considered as the RECK immunoreactivity for each subject. RECK immunoreactivity values greater than the median were considered as positive (or high expression).

### Cell lines and reagents

Six breast cell lines (S1, MCF-7, T47D, T4-2, MDA-MB-231 and Hs578T) with distinct tumorigenic and aggressiveness levels were used in this study [26]. The broad-spectrum MMP inhibitor (GM6001) was purchased from Millipore (Billerica, MA, USA).

### Viral transduction and shRNA experiments

For the inhibition of RECK expression, five different specific shRNA sequences (sequentially numbered from shRECK23 through shRECK27) were tested. These vectors and the structural ones belonged to the Mission® shRNA system (Sigma-Aldrich, St. Louis, MO, USA). Viral recombinant particles were generated by co-transfection of the 293FT virus packer cell line with these recombinant lentiviral vectors. Transduced cells were selected by the addition of 2 μg/mL puromycin.

### Quantitative RT-PCR studies

Total RNA was extracted from cell lines using the RNeasy Mini Kit (Qiagen, Germantown, MD, USA). Its integrity and quantity were evaluated using a NanoDrop™ 1000 Spectrophotometer (Thermo Fisher Scientific, Waltham, MA, USA). For cDNA synthesis, the SuperScript® III Reverse Transcriptase Kit was used (Life Technologies, Carlsbad, CA, USA). Quantitative RT-PCR was carried out using QuantiTect SYBR Green RT-PCR Kit (Qiagen). The primers used to amplify RECK, MMP-9 and the endogenous control (18S ribosomal RNA) were obtained from a validated primer panel available from Qiagen (QuantiTect Primer Assays). Each experiment was carried out in duplicate. The relative expression levels were calculated according to the 2^-∆∆Ct^ method [28].

### Western blotting

Total extracts were prepared from each cell culture line. Equal protein amounts from each extract were fractionated by SDS-PAGE and then electro-transferred to nitrocellulose membranes. After blockade with BSA, these membranes were incubated with RECK (Cell Signaling) and Lamin A/C (Santa Cruz, Santa Cruz, CA, USA) antibodies, diluted at 1:1000 and 1:200, respectively. Immunoreactive proteins were detected with the appropriate secondary horseradish peroxidase-conjugated antibody (GE Healthcare, Little Chalfont, UK) and visualized using the SuperSignal West Femto Chemiluminescent Substrate (Thermo Fisher Scientific). Quantitative densitometry of the electrophoretic band images was carried out using AlphaEaseFC software.

### Gelatin zymography assays

Gelatin zymography of the conditioned media was used to assess the *in vitro* enzymatic activity levels of MMP-9 in RECK-silenced and scrambled MDA-MB-231 cells. These samples were fractionated by 10 % SDS-polyacrylamide gel electrophoresis co-polymerized with the enzyme substrate, 0.1 % denatured type I collagen (gelatin type A; Sigma, St. Louis, MO, USA). After electrophoresis, the gels were washed at room temperature with 2.5 % Triton X-100, incubated for 48 h at 37 °C in substrate buffer containing 50 mM Tris buffer (pH 8.5) and 10 mM CaCl_2_. Gels were stained with Coomassie Blue R-250 (Sigma) and destained with 40 % methanol (Merck) and 10 % acetic acid (Merck) in water. Gelatinolytic activity was visualized as negatively staining bands. Each independent experiment was performed in duplicate. Quantitative densitometry of the electrophoretic band images was performed using the AlphaEaseFC software.

### *In vitro* invasion assays

To assess the role of RECK in cellular invasiveness potential, RECK-deficient or scrambled control MDA-MB-231 cells were plated on top of Matrigel-coated 8-μm-pore transwells (BD Bioscience, Franklin Lakes, NJ, USA). These cells were maintained in the presence of GM6001 or its vehicle (DMSO) in serum-free medium. The medium present in the bottom chamber was supplemented with 10 % bovine serum, used as a chemo-attractant. Cells were allowed to invade for 36 h. Cells remaining at the top chamber were removed, and those present at the bottom of the filter were stained and fixed with Coomassie Blue 0.125 % in methanol:acetic acid:H_2_O (45:10:45, v/v/v) for 15 min. Using the ImageJ® program, the relative cellular invasion was quantified from the images obtained (10× objective lens) under each experimental condition. Non-tumorigenic S1 mammary cells were used as a negative control. Triplicate wells were utilized per condition in each independent experiment.

### Statistical analysis

Statistical analysis of data from patients was performed using R (http://www.r-project.org/) [29]. Chi-square tests were used to assess the association between RECK immunoreactivity and different clinico-pathological parameters. P-values were corrected for multiple tests by the False Discovery Rate procedure [30]. Kaplan-Meier (KM) plots were used for overall (OS) and disease-free (DFS) survival analysis, and the log-rank test was used to compare curves separated according to RECK expression. For KM analysis, 3 of 1040 subjects were identified as being outliers by Tukey’s criterion; thus, they were removed from the data analysis. Cox proportional hazards regression was used to estimate hazard ratios (HRs). Subjects for whom no information on the analyzed covariates was available, were removed, with 940 of the 1040 subjects remaining for the Cox regression analysis.

Data obtained using the cell lines model were analyzed using the GraphPad Prism 5.0 program. In this case, statistical significance was determined using the one way ANOVA variance analysis and the Tukey-Kramer test. The results were presented as the mean ± standard error of the mean. A p-value less than 0.05 was considered to be statistically significant.

## Results

### RECK protein expression levels in human breast cells correlates with invasiveness

**Fig. 1:**
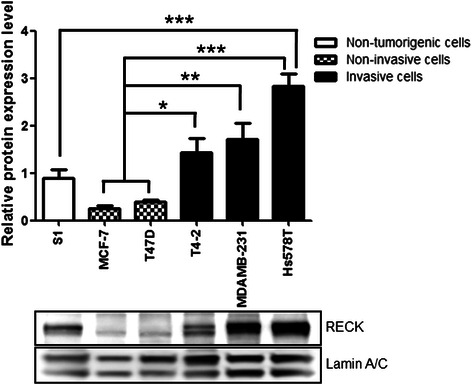
Invasive breast cancer cells express higher levels of RECK than non-invasive and non-tumorigenic ones. Western blot assays were performed to analyze RECK protein expression levels in a panel of human breast cells. The lamin A/C protein level was used as the loading control and for normalization. The results are presented as the means ± standard error from three independent experiments. *, *p* < 0.05, **, *p* < 0.01 and ***p < 0.001

In several tissue models, RECK expression was shown to be inversely related to oncogenesis [15, 31, 32], being expressed in normal tissues but not in many tumor cell lines and tumor samples [15, 32]. However, in breast cancer, the RECK expression profile remains uncertain and controversial [24–27]. To better understand this divergence, we quantified the RECK protein levels in a panel of breast cell lines with distinct aggressiveness levels, including non-tumorigenic (S1) cells, and non-invasive (MCF-7 and T47D) and invasive (T4-2, MDA-MB-231 and Hs578T) cancer cell lines (Fig. 1). We observed that more invasive cells (T4-2, MDA-MB-231 and Hs578T) displayed significantly higher (*p* < 0.05, *p* < 0.01 and *p* < 0.001, respectively) RECK protein levels than non-invasive breast cancer lineages (MCF-7 and T47D). Furthermore, RECK protein was significantly overexpressed in the highly invasive Hs578T cells (*p* < 0.001) compared with the non-malignant ones (Fig. 1). The doublet bands detected were possibly a result of RECK glycosylation [33]. Therefore, our results suggest that, unexpectedly, human breast cancer cells with a higher invasive potential express elevated levels of RECK (Fig. 1).

### RECK down-modulates invasion and MMP-9 in highly invasive breast cancer cells

**Fig. 2:**
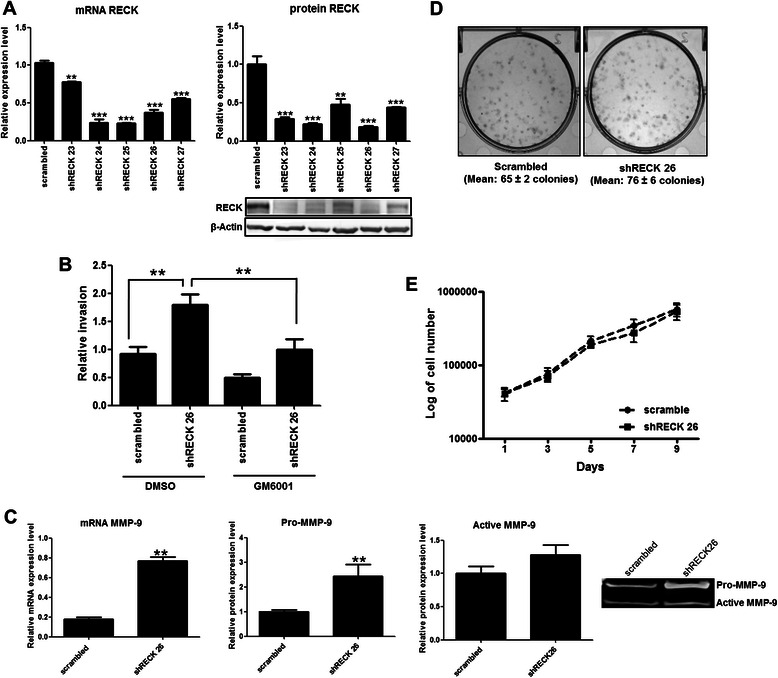
RECK functional analysis in the highly invasive MDA-MB-231 cells supports the role of RECK as an MMP inhibitor and negative modulator of cellular invasion. **a** RECK mRNA and protein expression levels in the MDA-MB-231 cells infected with lentiviral particles carrying different shRNA sequences specific for RECK inhibition (shRECK 23 through 27) or the scrambled control. The 18S ribosomal RNA expression and β-actin protein levels were used as endogenous controls in qRT-PCR and Western blot assays, respectively. **b** Transwell™ invasion assays were performed to assess the invasion capacity of scrambled control and RECK-deficient (shRECK26) cells that were treated with or without GM6001 (broad-spectrum MMP inhibitor). **c** MMP-9 mRNA and protein expression (pro-enzyme and active forms) levels in control (scrambled) and RECK-silenced cells quantified by qRT-PCR and Zymography assay, respectively. The 18S ribosomal RNA expression was used as the endogenous control in qRT-PCR assays. Proliferation analysis of scrambled and shRECK 26 cells by (**d**) colony formation assays and (**e**) growth curves. The results are presented as the means ± standard error from two independent experiments. **, *p* < 0.01 and ***, *p* < 0.001, all versus control (MDA-MB-231 scrambled cells)

To further clarify the specific role of RECK in mammary neoplasia, we evaluated the functional activity of RECK. To this end, MDA-MB-231 cells, a highly invasive human breast cancer cell line, were used as a study model. The RECK expression level in MDA-MB-231 cells was silenced using different shRNA sequences (shRECK oligos numbered 23 through 27). Their ability to silence RECK was confirmed by qRT-PCR and Western blot assays (Fig. 2a). All of the sequences tested could decrease RECK expression at both the mRNA and protein levels. Subsequent functional assays were performed using shRECK26-transduced cells in which RECK protein expression was reduced by 80 % relative to the scrambled control (Fig. 2a).

First, we addressed the role of RECK in the invasion of MDA-MB-231 cells. To this end, the *in vitro* invasion capacities of scrambled control and RECK-downmodulated cells were compared in Transwell™ assays (Fig. 2b). Our results indicate that the reduction of RECK led to a significant increase (*p* < 0.01) in MDA-MB-231 invasion. Treatment with a broad-spectrum MMP inhibitor (GM6001) suggests that this effect of RECK on the cell invasive capacity is largely MMP dependent because GM6001 could significantly (*p* < 0.01) reverse the RECK silencing effect in MDA-MB-231 cells (Fig. 2b). Additionally, we found that RECK-silenced cells express significantly higher levels of both MMP-9 (*p* < 0.01) mRNA and its pro-enzyme (*p* < 0.01) than scrambled control cells (Fig. 2c). Importantly, no influence of RECK was observed in MDA-MB-231 cell proliferation, as indicated in Fig. 2 (d and e). These results suggest that although its expression is correlated with increased breast cancer cell aggressiveness, RECK still serves as a negative regulator of cellular invasion and MMP-9 expression in MDA-MB-231 cells.

### RECK expression in breast tumor tissue samples and its association with clinico-pathological parameters

**Fig. 3:**
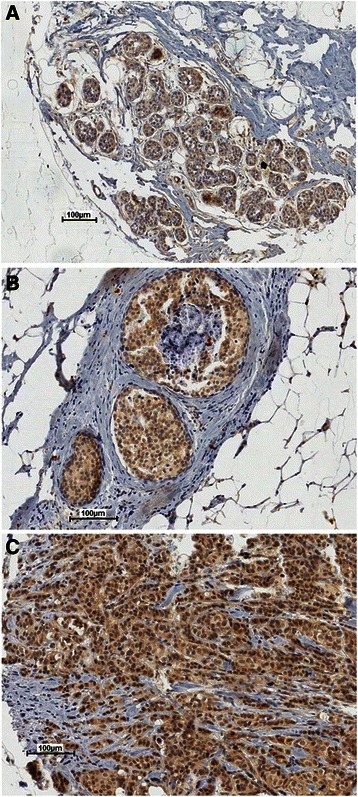
Representative immunohistochemistry images of the RECK staining profile detected in breast tissue samples show RECK expression in epithelial cells. **a** Benign proliferation, (**b**) ductal carcinoma *in situ* (DCIS) and (**c**) invasive tumor samples analyzed in tissue microarray cores

Previous reports have supported the potential role of RECK as a molecular marker for the prognosis of several cancer types (23). However, the association between RECK expression and the outcome of breast cancer patients remains unclear. We evaluated RECK protein expression levels in a large series (*n* = 1040) of breast cancer cases by immunohistochemistry on TMAs. The staining pattern obtained for RECK in mammary tissue samples derived from benign proliferation, ductal carcinoma *in situ* (DCIS) and invasive tumors is presented in Fig. 3. In all of these distinct breast tissues, RECK was predominantly expressed in epithelial cells, while the stromal cells were negative.

All of the breast tumor samples included in this analysis were classified as positive or negative for RECK considering the intensity and percentage of stained cells (Methods). To evaluate the correlation between RECK expression and different clinico-pathological parameters, Chi-squared tests were performed (Table 1). Except for histological grade (*p* = 0.0008) and TNM stage (*p* = 0.0114), there was no correlation between RECK and most of the analyzed factors (age, menopausal status, tumor size, lymph node stage, recurrence and ER/PR status).

**Table 1:** Association between RECK expression and clinicopathological parameters

Parameter	RECK negative no. cases (%)	RECK positive no. cases (%)	*χ*2	p-value
Age				
< 50 years	195 (18.8 %)	191 (18.4 %)	0.021	0.8848
≥ 50 years	324 (31.3 %)	326 (31.5 %)		
Menopausal status				
Premenopausal	206 (20.1 %)	197 (19.1 %)	0.082	0.7752
Postmenopausal	312 (30.4 %)	312 (30.4 %)		
Tumor size				
< 5 cm	252 (25.5 %)	268 (27.1 %)	1.494	0.2216
≥ 5 cm	247 (24.9 %)	223 (22.5 %)		
Lymph node stage				
Negative (N0)	169 (16.6 %)	160 (15.7 %)	0.243	0.6222
Positive (N1, N2, N3)	341 (33.5 %)	348 (34.2 %)		
Recurrence				
Negative	257 (25.1 %)	259 (25.3 %)	0.063	0.8014
Positive	258 (25.2 %)	250 (24.4 %)		
ER/PR status				
ER+/PR+	208 (22.3 %)	204 (22.0 %)	8.001	0.046
ER+/PR-	125 (13.5 %)	86 (9.3 %)		
ER-/PR+	8 (0.9 %)	11 (1.2 %)		
ER-/PR-	135 (14.5 %)	151 (16.3 %)		
Histological grade				
1	86 (8.3 %)	73 (7.1 %)	14.257	**0.0008**
2	315 (30.5 %)	273 (26.4 %)		
3	116 (11.2 %)	170 (16.5 %)		
TNM stage				
1	39 (3.8 %)	17 (1.6 %)	11.069	**0.0114**
2A and 2B	201 (19.5 %)	204 (19.7 %)		
3A, 3B, and 3C	240 (23.2 %)	240 (23.2 %)		
4	39 (3.8 %)	54 (5.2 %)		

*χ*2, Chi-square test coefficient; ER/PR, estrogen receptor/progesterone receptor

p-values considered significant after FDR correction were highlighted in bold

### RECK is not a prognostic indicator for breast cancer patients

**Fig. 4:**
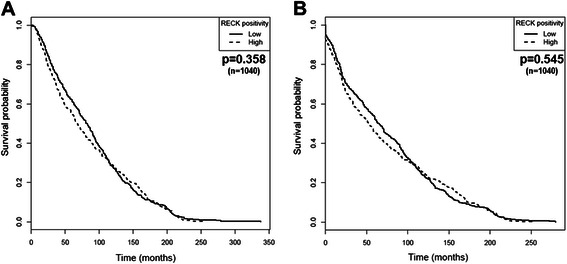
RECK does not a predictive value for the survival of breast cancer patients. Differences in the overall (**a**) and disease-free survival (**b**) based on RECK immunoreactivity intensity of the diagnosed tumors were assessed using Kaplan-Meier plots

To determine whether RECK would be a useful marker for the survival rate or could serve as a prognostic indicator, we performed several sets of analyses. The association between RECK and the overall (OS) and disease-free (DFS) survival was analyzed using Kaplan-Meier curves (Fig. 4). RECK expression did not predict any significant differences in OS and DFS (Fig. 4) among all of the 1040 breast cancer cases analyzed.

**Fig. 5:**
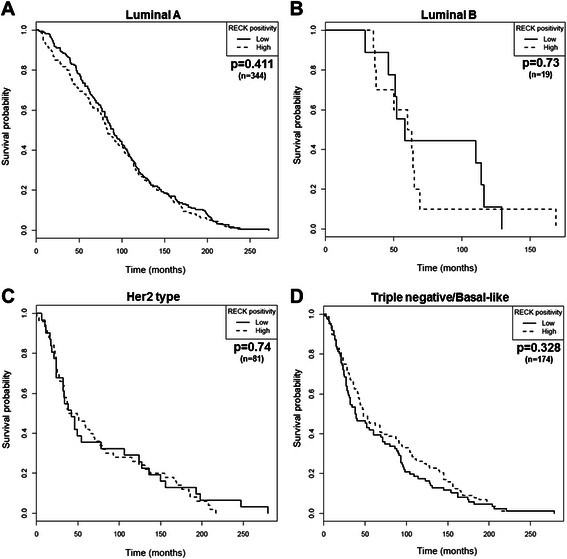
RECK is not associated with the overall survival (OS) of breast cancer patients regardless of the molecular subtype examined. Kaplan-Meier plots were obtained for OS analysis stratified by RECK expression in patients diagnosed with (**a**) luminal A, (**b**) luminal B, (**c**) Her2 type and (**d**) basal-like breast tumors

**Fig. 6:**
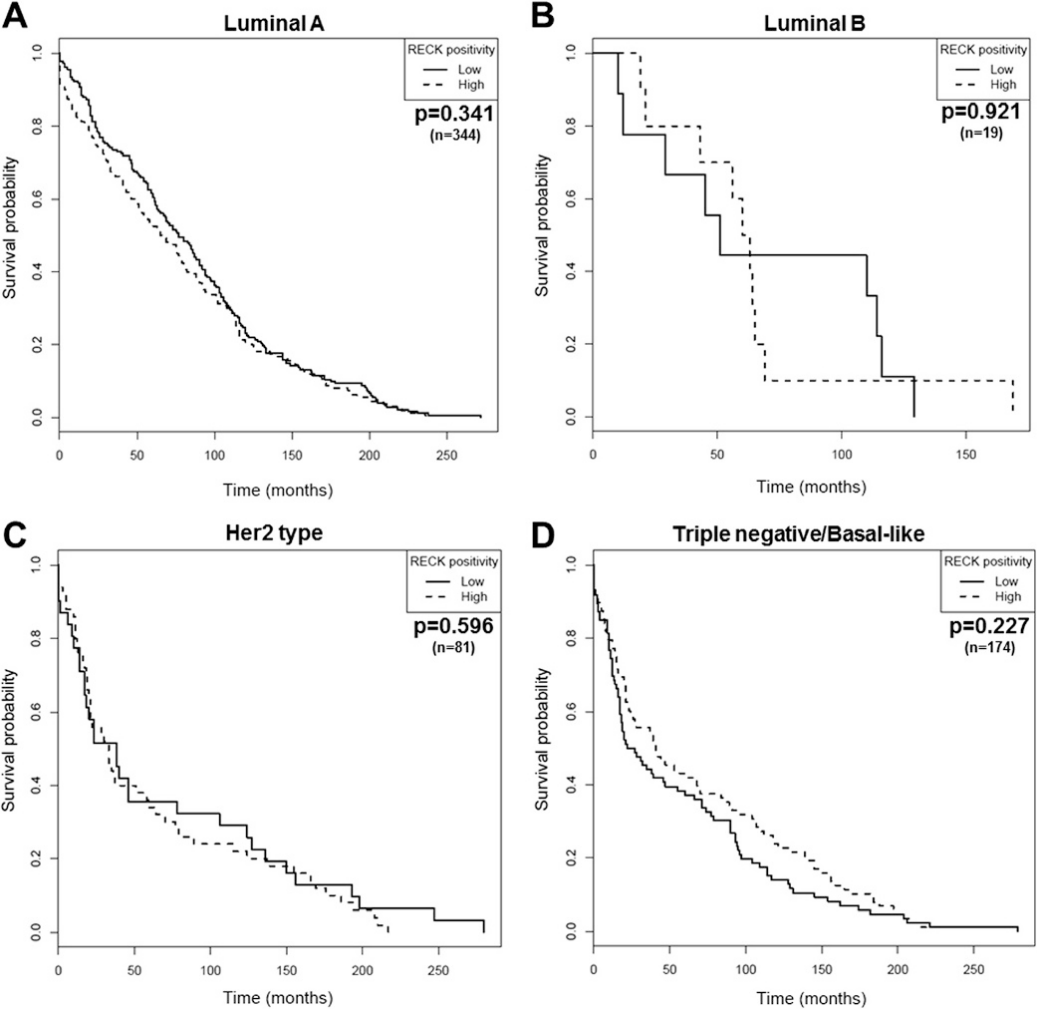
RECK is not a prognostic marker of disease-free survival (DFS) for patients diagnosed with different breast cancer molecular subtypes. Kaplan-Meier plot estimate of DFS stratified by RECK expression in breast cancer patients diagnosed with (**a**) luminal A, (**b**) luminal B, (**c**) Her2 type and (**d**) basal-like tumors

The prognostic significance of RECK for specific cohorts of patients was further evaluated. The tumor samples were classified according to molecular subtypes, namely luminal A, luminal B, Her2 type and basal-like/triple negative, which, in turn, were determined by the status of crucial breast cancer biomarkers (ER, PR and Her2). Samples were excluded from the analysis once the parameters required for subtype discrimination were not available. Thus, the predictive value of RECK was tested in 344 cases of luminal A, 19 cases of luminal B, 81 cases of Her2 type and 174 cases of Triple-negative/basal-like breast cancer. Regardless of each molecular subtype examined, differences in the RECK protein levels were not associated with significant changes in OS (Fig. 5) or DFS (Fig. 6).

**Fig. 7:**
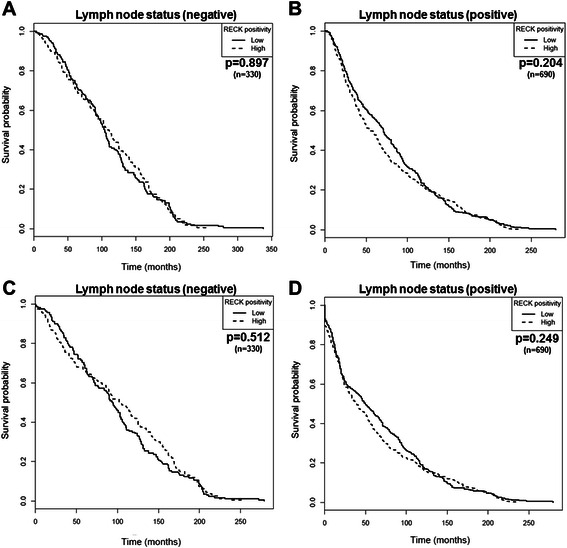
RECK is not related to survival of breast cancer patients regardless of the lymph node status. Kaplan-Meier curves of overall (**a** and **b**) and disease-free (**c** and **d**) survival stratified by RECK expression in breast cancer patients classified as lymph node negative (**a** and **c**) or positive (**b** and **d**) at diagnosis

The presence of tumor cells in axillary lymph nodes is also critical for the staging and prognosis of breast cancer, in addition to providing a more assertive therapy choice. Survival analyses were also performed considering two distinct groups: lymph node-negative (*n* = 330) or -positive (*n* = 690) patients. However, independently of lymph node status, RECK remained unable to predict the OS and DFS of these patients (Fig. 7). Multivariate Cox regression analysis demonstrated that RECK did not provide independent prognostic information for breast cancer patient survival (Tables 2 and 3), strengthening the already reported evidence that this MMP inhibitor is not a relevant biomarker for breast cancer.

**Table 2:** Cox regression for overall survival analysis in the entire patient cohort

	Entire cohort (*n* = 940)					
	Univariate			Multivariate^a^		
Prognostic factor	HR	95 % CI	p-value	HR	95 % CI	p-value
RECK (low *vs.* high)	0.95	0.67–1.37	0.8	0.91	0.63–1.31	0.63
Age (continuous)	1.01	1.01–1.02	<0.01	1.01	1.01–1.02	<0.01
Tumor size (continuous)	1.05	1.02–1.07	<0.01	1.03	1.01–1.06	0.02
Histological grading (3 *vs.* 1–2)	1.27	1.14–1.40	<0.01	1.24	1.11–1.38	<0.01
TNM stage (IV *vs.* I–III)	1.05	0.98–1.13	0.15	1	0.93–1.07	0.96
Nodal status (positive *vs.* negative)	1.53	1.34–1.76	<0.01	1.51	1.32–1.74	<0.01
ER (positive *vs.* negative)	0.88	0.77–1.01	0.08	0.96	0.81–1.13	0.6
PR (positive *vs.* negative)	0.88	0.77–1.00	0.05	0.96	0.82–1.12	0.59
Her2 (positive vs. negative)	1.22	1.01–1.48	0.04	1.01	0.82–1.25	0.91

RECK. REversion-inducing Cysteine-rich protein with Kazal motifs; ER. estrogen receptor; PR. progesterone receptor; Her2. human epidermal growth factor 2

^a^Adjusted for all other variables in the table

**Table 3:** Cox regression for disease-free survival analysis in the entire patient cohort

	Entire cohort (*n* = 940)					
	Univariate			Multivariate^a^		
Prognostic factor	HR	95 % CI	p-value	HR	95 % CI	p-value
RECK (low *vs.* high)	0.98	0.69-1.38	0.89	0.95	0.64–1.31	0.63
Age (continuous)	1.01	1.00–1.01	<0.01	1.01	1.00–1.02	<0.01
Tumor size (continuous)	1.08	1.06–1.11	<0.01	1.06	1.03–1.09	<0.01
Histological grading (3 *vs.* 1–2)	1.28	1.16–1.42	<0.01	1.26	1.13–1.41	<0.01
TNM stage (IV *vs.* I–III)	1.13	1.05–1.22	<0.01	1.07	0.99–1.14	0.08
Nodal status (positive *vs.* negative)	1.57	1.37–1.80	<0.01	1.51	1.31–1.74	<0.01
ER (positive *vs.* negative)	0.89	0.78–1.02	0.11	1.04	0.87–1.23	0.68
PR (positive *vs.* negative)	0.85	0.75–0.97	0.01	0.92	0.79–1.08	0.3
Her2 (positive vs. negative)	1.16	0.96–1.40	0.13	0.96	0.78–1.18	0.71

RECK. REversion-inducing Cysteine-rich protein with Kazal motifs; ER. estrogen receptor; PR. progesterone receptor; Her2. human epidermal growth factor 2

^a^Adjusted for all other variables in the table

## Discussion

Since its first report, RECK down-regulation has been linked to tumor progression [15, 32], and it is considered to be an adequate biomarker for a better clinical course for patients diagnosed with various tumors [23]. Nevertheless, in this study, we obtained surprising evidence that RECK is not associated with breast cancer patient survival. Initially, our results may seem contradictory to previously published data. However, despite the extensive amount of work addressing the RECK prognostic value for prostate, lung and pancreatic tumors, RECK function and expression profile in breast cancer remain an open question. The few preceding papers on human mammary gland assessed RECK in a reduced number of samples and, in most cases, only at the transcriptional level [24–27]. Moreover, these prior studies are controversial. Some of them corroborate the typical RECK down-modulation as a hallmark of tumor aggressiveness [24, 25]. Conversely, another report demonstrated not only higher RECK levels in invasive breast cancer cell lines than in the non-invasive one but also a positive correlation between RECK and MMP expression in tumor tissue samples [26]. In the present work, we measured RECK protein levels both in a panel of human breast cell lines and in a large series (1040) of breast cancer cases, allowing a more significant conclusion.

Additionally, we performed a functional analysis of RECK in a breast cancer cell model. Despite the differential expression profile displayed by RECK in breast cancer samples, we confirmed that it performs similar functions in MDA-MB-231 cells to those previously described in other models. In this cell culture model, RECK continues to act as an invasion inhibitor via MMP down-modulation even in highly invasive human breast cancer cells. Although, at first, it may seem conflicting, we hypothesize that, to restore the balance between proteases and their inhibitors, cells respond by inducing RECK. In fact, an increase in both MMPs and TIMPs has been described as a central and crucial hallmark of tumor progression. Although this may be a mechanism to maintain the protease/inhibitor ratio in balance, this cellular response mechanism cannot block disease progression.

## Conclusions

In contrast to other tumor types, RECK expression cannot predict breast cancer patient survival, although its inhibitory function in invasion has been confirmed. Despite the well-known cancer heterogeneity, several studies are still aiming to identify a universal tumor marker and draw an overall conclusion. However, in agreement with the emerging concept of personalized cancer therapy, general conclusions should be avoided, and more tumor-specific biomarkers should be sought.

## Abbreviations

CK: Cytokeratin
DFS: Disease-free survival
ECM: Extracellular matrix
ER: Estrogen receptor
KM: Kaplan-Meier
MMP: Matrix metalloproteinase
OS: Overall survival
PR: Progesterone receptor
qRT-PCR: Quantitative reverse transcription polymerase chain reaction
RECK: Reversion-inducing cysteine-rich protein with Kazal motifs
TIMP: Tissue inhibitors of matrix metalloproteinases
TMA: Tissue microarray
